# In Vivo and In Vitro Virulence Analysis of Four Genetically Distinct *Toxoplasma gondii* Lineage III Isolates

**DOI:** 10.3390/microorganisms8111702

**Published:** 2020-10-31

**Authors:** Aleksandra Uzelac, Ivana Klun, Vladimir Ćirković, Olgica Djurković-Djaković

**Affiliations:** Center of Excellence for Food- and Vector-borne Zoonoses, Institute for Medical Research, University of Belgrade, 11129 Belgrade, Serbia; aleksandra.uzelac@imi.bg.ac.rs (A.U.); iklun@imi.bg.ac.rs (I.K.); vladimir.cirkovic@imi.bg.ac.rs (V.Ć.)

**Keywords:** *Toxoplasma gondii*, virulence, lineage III variants, proliferation, metabolism

## Abstract

*Toxoplasma gondii* archetypes II and III are mildly virulent, yet virulence of variant strains is largely unknown. While lineage II dominates in humans in Europe, lineage III strains are present in various intermediate hosts. In Serbia, lineage III represents 24% of the population structure and occurs most frequently in domestic animals, implying a significant presence in the human food web. In this study, the virulence of four genetically distinct lineage III variants was assessed in vivo and in vitro. In vivo, two strains were shown to be intermediately virulent and two mildly virulent, with cumulative mortalities of 69.4%, 38.8%, 10.7%, and 6.8%, respectively. The strain with the highest mortality has previously been isolated in Europe and may be endemic; the strain with the lowest mortality matches ToxoDB#54, while the remaining two represent novel genotypes. Identical alleles were detected at ROP5, ROP16, ROP18, and GRA15. A set of in vitro analyses revealed proliferation and plaque formation as virulence factors. Higher levels of expression of ENO2 in intermediately virulent strains point to enhanced metabolism as the underlying mechanism. The results suggest that metabolic attenuation, and possibly stage conversion, may be delayed in virulent strains.

## 1. Introduction

*Toxoplasma gondii*, a protozoa of the phylum Apicomplexa, is a globally distributed food and waterborne parasite of all homeothermic species and whose definitive hosts are Felidae [1]. The parasite has three distinct life stages: the rapidly proliferating motile tachyzoite capable of infecting all nucleated cells, the metabolically less active bradyzoite, and the sporozoite, which forms in the oocysts excreted by the definitive host [2]. Infection is initiated following invasion of host cells by tachyzoites, which rapidly proliferate by endodyogeny and then disseminate to colonize various tissues. As the tachyzoite is vulnerable to the host’s immunity mediated defenses, persistence of the parasite in any infected host is enabled by conversion into bradyzoites and biogenesis of double membraned protective cysts located predominantly in neurons and myocytes of skeletal muscles and the heart. These cell types seem to promote encystation and, importantly, increase the chance of transmission (myocytes) by ingestion, or are particularly chemically attractive to tachyzoites (neurons) [3]. While there is only one species of *T. gondii*, a number of different genotypes have been identified that belong to different lineages. Three major lineages circulate globally (types I, II, III) along with several mostly localized lineages, such as haplotype 12, Africa 1, Africa 3, and Brazil I-IV [4]. Genotypes known as archetypes, with identical allele types at all 11 canonical loci used for MnPCR-RFLP genotyping, have been identified only for the three major lineages, while variants, which can carry a combination of allele types, occur in all lineages. Sustained survival of the species necessitates continuous evolution of new genotypes, which are primarily driven by recombination of different genomes and the formation of haploid sporozoites by meiosis. Oocysts, resistant to significant and prolonged physical and chemical stresses of the environment into which they are shed [5], grant survival of sporozoites with possibly novel genotypes. The parasite is infectious during all three life stages and ingested bradyzoites (within tissue cysts) and sporozoites (within oocysts) spontaneously convert into tachyzoites in all hosts. 

The global prevalence of human toxoplasmosis varies remarkably, both within and between countries, with the vast majority of cases resulting from infections acquired by ingestion of tissue cysts [6] and oocysts via food and water [7]. Minor modes of transmission include vertical transmission of tachyzoites from the mother to the fetus, resulting in congenital infection, and horizontal transmission as a consequence of transplantation and/or transfusion [2]. In immunocompetent people, the infection is most often asymptomatic or accompanied by mild flu-like symptoms, which at present, is still believed to resolve without sequelae, despite concerns raised by a formidable body of research, which aims to associate infection followed by persistence of tissue cysts in the brain with neuropsychiatric disorders, notably schizophrenia [8]. Clinically, *T. gondii* is an important opportunistic pathogen as the infection can be life threatening or even lethal for the immunocompromised [9]. In addition to the immune status of the individual host, it has been experimentally established that the severity of the clinical presentation and disease outcome correlates with the virulence of the parasite’s genotype, which it certainly does for mice [10,11,12]. Thus, lineage I archetype is the most virulent, defined as lethal to mice following inoculation of even a single tachyzoite, while virulence of the lineage II and III archetypes may range between low and mild, in a dose-dependent manner. While archetypes have been extensively used experimentally, the virulence of variant genotypes of the three global as well as a multitude of local lineages is mostly unknown and, as research has shown, rather difficult to predict based on genetics alone. Population genetics in combination with clinical experience indicate that in Europe, where lineage II strains, particularly the archetype, are dominant and the diversity of the strain population is low, clinical symptoms are primarily mild, which in turn suggests that virulent genotypes are rare [13,14]. However, in South America, where the three major lineages circulate along with much more abundant genotypes of exotic autochthonous lineages and diversity of the strain population is high, clinical symptoms are often severe, indicating that virulent genotypes are common [4,12]. In fact, it has been suggested to recognize a new clinical entity named Amazonian toxoplasmosis, because of its uncharacteristically severe symptomatology and possible lethal outcome [15]. Concordantly, evaluating the virulence of individual strains and identifying the underlying mechanisms has become crucial in understanding host pathology in toxoplasmosis.

**Figure 1:**
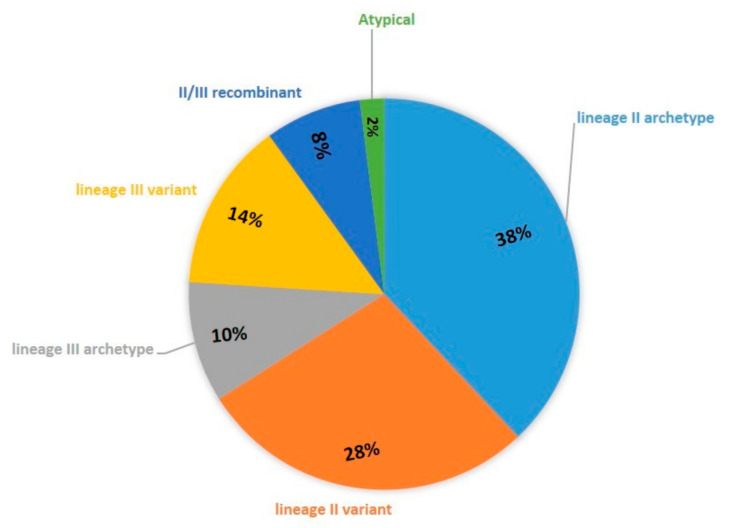
Population structure of *T. gondii* in Serbia (*n* = 50 strains).

Over the years, different genotypes were detected and/or isolated from intermediate hosts in Serbia [16,17,18,19,20,21]. Recently, the population structure of *T. gondii* was determined [22] based on 50 strains from humans, domestic animals, and wild canids (Figure 1). As expected, archetype II dominates, comprising 38% of the total structure, while variants make up 28%. Lineage III strains were detected in domestic animals and wild canids, indicating circulation in both domestic and sylvatic environments, with the archetype representing 10% of the population structure, variants 14% and II/III recombinant strains 8%. The virulence of lineage III variants warrants analysis, as they represent a significant component of the parasite’s population structure in Serbia and occur in food animals. Here, we present the comparative virulence analysis of four genetically distinct lineage III variants.

## 2. Materials and Methods

### 2.1. Study Design

The virulence of four lineage III strains—of which one was isolated in 2013 from heart tissue of a feral pigeon (G13) from the highly urban territory in and around the capital Belgrade [18], two were isolated in 2016 from domestic horses raised in rural regions in western Serbia (EQ39, EQ40) [19], and one was isolated in 2017 from a domestic free range chicken (K1) from the suburbs of Belgrade [21] genotyped by MnPCR-RFLP—was determined in vivo by the mouse survival assay as described by Saraf et al., 2017 [23]. Possible virulence mechanisms were examined, including allele typing of four virulence markers ROP5, ROP16, ROP18, and GRA15 [24], in vitro growth and plaque assays [25] and the relative quantification of ENO2, the tachyzoite specific enolase involved in glycolysis [26]. The strains were isolated and maintained by passage twice a year in Swiss Webster mice and transferred to cell culture only for the duration of the in vitro experiments. 

### 2.2. Genotyping and Virulence Marker Typing by MnPCR-RFLP

Genotyping was performed using conventional markers Alt.SAG2, GRA6, BTUB, C-22, PK1, CS3, L358, C29-2, and APICO, while each multiplex PCR reaction and subsequent nested reaction was prepared, as previously described in Su et al., 2010 [27]. Briefly, all reactions were run in a final volume of 20 µL, which contained PCR MasterMix (Thermo Fischer Scientific, Waltham, MA, USA), 0.15 µM of the external forward and reverse primers for all markers, or 0.3 µM of internal forward and reverse primer for each marker and 2 µL of gDNA as template. The thermal cycling program for the multiplex reaction was 3 min at 95 °C for initial denaturation, 30 cycles of 30 s at 95 °C, 60 s at 55 °C, 60 s at 72 °C, and a final extension for 5 min at 72 °C. For the nested reactions, initial denaturation and final extension remained the same, but with 35 cycles of 95 °C for 30 s, 60 °C for 60 s, and 72 °C for 60 s for all markers except that the annealing temperature for APICO was 58 °C. For allele typing of virulence markers ROP5, ROP16, ROP18, and GRA15 the same approach was used, as described previously in Dubey et al., 2014 [24], except that each reaction contained 0.25 µM of the external forward and reverse primers and 0.5 µM of the internal forward and reverse primers. PCR was performed in the Veriti thermal cycler (Applied Biosystems, Foster City, CA, USA). RFLP was performed using appropriate restriction enzymes for each product and a digestion mixture consisting of 1–2 U of restriction enzyme, 1X Fast Digest (FD) buffer (Thermo Fisher Scientific, Waltham, MA, USA) and 5 µL of the Mn-PCR product in a final volume of 25 µL. RFLP products were separated by electrophoresis in 2.5% agarose gels stained with ethidium bromide and visualized in a BioDocAnalyze instrument (Biometra, Göttingen, Germany). gDNA of strains RH (archetype I), Me49 (archetype II), and NED (archetype III) were used as RFLP standards.

### 2.3. Mice

Swiss Webster female mice weighing between 18–20 g were purchased from the Animal Research Facility of the Military Medical Academy in Belgrade, Serbia and transferred into the Institute for Medical Research Animal Research Facility. Mice were housed 5 per cage, kept at a natural light cycle and offered food and water ad libitum. Prior to any experimental procedure, the animals were allowed to rest and acclimate for a week. The cages were cleaned and the bedding was changed at least three times a week. 

All animal experiments were approved by the Ethics Council of the Ministry of Agriculture, Forestry and Water Management of Serbia Veterinary Directorate (Decision no. 323-07-05567/2019-05 of 10 July 2019). 

### 2.4. In Vivo Conversion of Bradyzoites to Tachyzoites and Tachyzoite Propagation in Cell Culture

Swiss Webster mice were infected intraperitoneally (i.p.) with 5 tissue cysts of each *T. gondii* strain, obtained from chronically infected animals, suspended in sterile saline fluid (Hemofarm A.D., Vrsac, Serbia) supplemented with gentamycine (1.6 mg/kg) in a final volume of 500 µL. Mice were sacrificed by cervical dislocation 5 days post infection (p.i.), and the peritoneal fluid, which contained various cells infected with tachyzoites, was removed using a 5 mL syringe with an 18 G hypodermic needle. The peritoneal fluid was centrifuged in an Eppendorf 5702 (Eppendorf, Hamburg, Germany) swinging bucket centrifuge at 1800 rpm for 8 min, then washed with 3 mL of saline fluid and centrifuged again. The pellet was re-suspended in 200 µL of DMEM medium (ThermoFisher Scientific, Waltham, MA, USA) with 2% bovine calf serum (Sigma-Aldrich, St. Louis, MO, USA) and inoculated into previously prepared Vero cell cultures in T25 culture flasks at 30–40% confluency. The cultures were monitored daily. When large numbers of the Vero cells were successfully colonized (usually within 24 h), the culture medium was replaced to remove any remaining murine cells. After 4–5 days of incubation, the tachyzoites were harvested. Infected Vero cells were detached from the flask using a cell scraper (Grainer Bio-One, Kremsmünster, Austria), and the culture medium was collected and centrifuged, as described above. The pellet containing infected Vero cells and extracellular tachyzoites was washed twice with sterile dPBS (ThermoFisher Scientific, Waltham, MA, USA) and finally resuspended in 1 mL. The suspension was passed through an 18 G hypodermic needle several times (4–5) to break up the cells and release intracellular tachyzoites. Finally, an aliquot of the suspension was removed and stained with Trypan blue for counting in a Burker-Turk hemocytometer. The obtained tachyzoites were either used for experimental procedures or re-inoculated into fresh Vero cell cultures with 30–40% confluency.

### 2.5. In Vivo Survival Assay

The in vivo survival assay was set up according to previously published protocols [23]. Briefly, Swiss-Webster female mice were infected i.p. with 500 µL suspensions containing serial dilutions of tachyzoites from 10^1^–10^4^/mouse in sterile saline (Hemofarm A. D., Vršac, Serbia) supplemented with gentamycine (1.6 mg/kg). Survival was monitored daily and mortality recorded for 42 days. The experiment was repeated three times for each strain. Deceased animals were promptly removed from the cages and the bedding was changed as soon as possible, while severely ill animals (with symptoms including hemiparesis, severe lethargy, refusal to eat, etc.) were sacrificed using cervical dislocation to prevent excessive suffering. All surviving animals were tested by the modified agglutination test (MAT) at day 28 post infection to confirm that they are infected and their eligibility for inclusion in the determination of cumulative mortality. The MAT, a slightly modified version of the agglutination test introduced by Desmonts and Remington, 1980 [28], was performed as previously described in Klun et al., 2017 [19] using an in house antigen. Infection in animals deceased prior to day 28 was confirmed by detection of the 529 bp repeat element by real time PCR from blood and/or brain tissue, as previously described [21,29]. Briefly, DNA was extracted from either 100 µL of whole blood obtained by cardiac puncture or 100 µL of brain tissue homogenized with sterile saline, using the DNeasy Blood and Tissue kit (Qiagen, Hilden, Germany) according to the manufacturer’s protocols. Blood was taken from severely ill animals prior to sacrifice, while the brain was harvested from deceased animals. *T. gondii* DNA was detected using a specific in house validated Taqman probe (Invitrogen, Carlsbad, CA, USA) in a total reaction volume of 20 µL, which contained 10 µL of Maxima Probe/ROX qPCR Mastermix (ThermoFisher Scientific, Waltham, MA, USA), 7.5 mM of forward and reverse primers, and DNase and RNase free water. DNA from the *T. gondii* RH strain was used as the positive control, while DNase and RNase free water was used as the negative control for each reaction batch. Real time PCR was performed in a Step One Plus thermal cycler (Applied Biosystems, Foster City, CA, USA) using a two-step end-point detection setup, with an initial hot start of 15 min at 95 °C, followed by 45 cycles of denaturation at 95 °C for 15 s and annealing/extension at 60 °C for 60 s.

### 2.6. In Vitro Growth and Plaque Assay

The growth assay protocol was performed as previously described [25]. Vero cells were seeded into 24-well plates (50,000 cells/well) with DMEM medium (ThermoFisher Scientific, Waltham, MA, USA) containing 2% bovine calf serum (Sigma-Aldrich, St. Louis, MO, USA) and allowed to adhere for at least 2 h. Cells were infected with tachyzoites of different strains in triplicate at a multiplicity of infection (MOI) of 0.1. Invasion was ascertained visually (presence/absence of infected cells) by inspecting at least 500 cells/strain starting at 1 h post infection in several intervals up to 3 h post infection. Cells were then vigorously washed with sterile dPBS (ThermoFisher Scientific, Waltham, MA, USA) to remove any extracellular tachyzoites and incubated at 37 °C with a 5% CO_2_ atmosphere. Parasites inside vacuoles were counted at 48 h post infection, and the number of divisions was estimated as log_2_ of the number of parasites per vacuole. A minimum of 30 vacuoles were counted per strain, and each experiment was repeated five times. 

For the plaque assay, confluent Vero cell cultures in 24-well plates were infected with tachyzoites of different strains at an MOI of 0.5 and incubated at 37 °C with a 5% CO_2_ atmosphere for 10 days. The growth medium was changed once at day 5. After 10 days, the wells were stained with 1% crystal violet solution, and plaques were counted. Three replicate wells were analyzed for each strain, and the experiments were repeated three times.

### 2.7. RNA Extraction, Reverse Transcription, and Relative Quantification

Vero cells were seeded into 24-well plates and infected with tachyzoites of the individual strains in triplicate at an MOI of 0.5, as previously described for the plaque assay. The plate was incubated as previously described for 10 days, with one growth medium change on day 5. After 24 h and on day 10, the medium was removed, each well was washed twice with dPBS (ThermoFisher Scientific, Waltham, MA, USA), and the dPBS was removed. The three replicate wells for each strain at each time point were rinsed consecutively with 1 mL of Trizol reagent (Invitrogen, Carlsbad, CA, USA) to lyse, harvest, and pool the contents. Extraction of total RNA was performed according to the manufacturer’s instructions. The total RNA yield was quantified using the Qubit RNA BR (broad range) kit and the Qubit 2.0 fluorimeter (ThermoFisher Scientific, Waltham, MA, USA) according to the manufacturer’s instructions. An amount of 500 ng of first strand cDNA was synthesized from each sample using the RevertAid First Strand cDNA Synthesis kit (ThermoFisher Scientific, Waltham, MA, USA) according to the manufacturer’s protocol, except that the random hexamer primer was replaced with OligoDT to capture mRNA only. Relative quantification was performed using β-actin as the normalizer (forward primer: 3′-TCCCGTCTATCGTCGGAAAG-5′, reverse primer: 3′-CCATTCCGACCATGATACCC-5′) [30] and ENO2 forward primer: 3′-CCATCAGGACATCACTGC-5′ and ENO2 reverse primer: 3′-GTTATCAAGGACATCGTTGCACG-5′ [26]. Each reaction contained 10 µL of PowerUp SYBR Green master mix (Applied Biosystems, Foster City, CA, USA), 10 pmol of each primer, 2 µL of cDNA template, and DNAse and RNAse free water in a final volume of 20 µL. The reactions were run in duplicate. cDNA synthesized from mRNA of the RH strain tachyzoites retrieved from the peritoneal cavity of Swiss Webster mice 3 days post infection was used to calibrate the expression of ENO2 at both time points. The total RNA isolation and cDNA synthesis was performed as described above. The relative quantity was calculated using the ΔΔCt method with kinetic PCR efficiency correction and calibration [31].

### 2.8. Statistical Analyses

Graphs were generated and statistical analyses were performed using the GraphPad Prism8 and Excel software. The Mantel–Cox test was used for the log rank comparison of the cumulative survival curves, while two-tailed Student’s t-tests were used to compare vacuole loading and plaque number.

## 3. Results

MnPCR-RFLP genotyping of the four isolates analyzed showed that EQ40, K1, and G13 are distinct lineage III variant strains, while EQ39 is a II/III recombinant strain (Table 1). The genotype of isolate EQ39 matches ToxoDB#54, while the other isolates could not be matched in ToxoDB (www.toxodb.org/toxo/app). 

**Figure 2:**
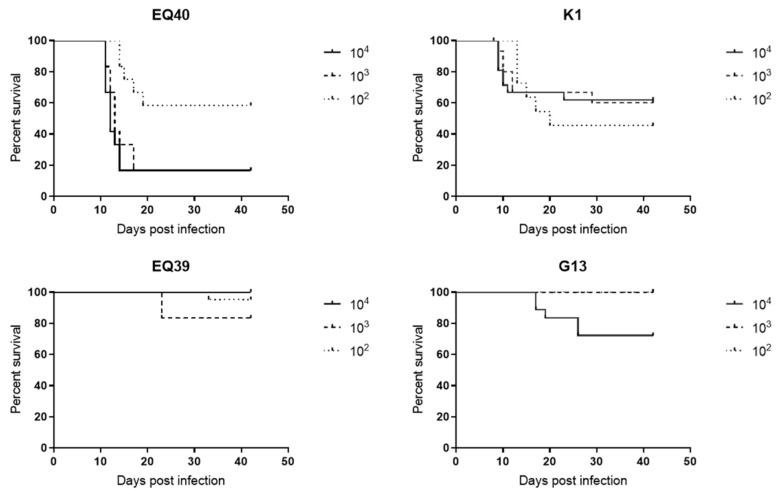
Survival curves of Swiss–Webster mice infected with different doses of tachyzoites of the lineage III isolates.

Infection of Swiss Webster mice with these strains using four different doses of tachyzoites (10^1^–10^4^) for in vivo virulence estimation resulted in dramatically different survival rates and revealed that EQ40 and K1 cause significant mortality, while EQ39 (ToxoDB#54) and G13 do not (Figure 2). Log rank comparison of the cumulative survival curves using the Mantel–Cox test confirmed that they were significantly different (*p* < 0.0001). In comparison, the lineage II archetype strain BGD18 did not cause any mortality in this experimental setup (data not shown). Since not all mice were infected with the lowest infective dose (10 tachyzoites), we have calculated the cumulative mortalities from survival to day 42 based on three consecutive doses from 10^2^ to 10^4^. The results classified EQ40 and K1 as intermediately virulent and EQ39 (ToxoDB#54) and G13 as non-virulent according to the criteria in Su et al., 2002 [32] (Table 2). Among all animals that succumbed to the infection, a large majority of mice infected with EQ40 and K1 died up to day 15 (88% and 76.1%, respectively), when the mortality for EQ39 and G13 was virtually null. 

Allele typing of virulence markers ROP5, ROP18, ROP16, and GRA15 yielded identical results for all four lineage III isolates, with type 1 allele for ROP16 and type 3 alleles for ROP5, ROP18, and GRA15 (Table 3). 

**Figure 3:**
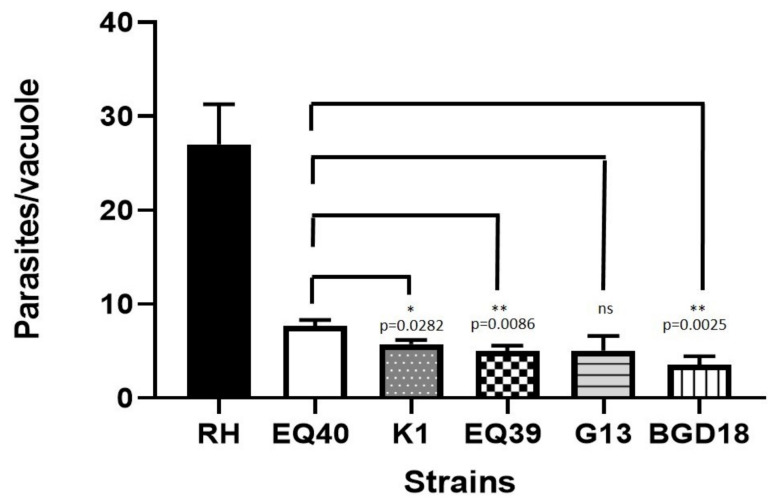
Mean vacuole loading after 48 h (* *p* < 0.05; ** *p* < 0.01).

**Figure 4:**
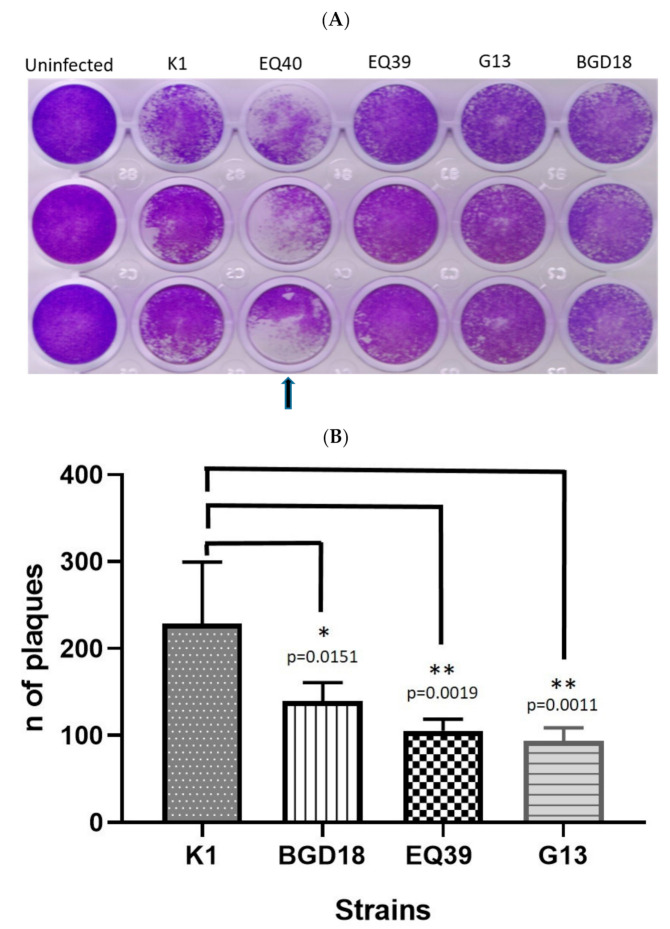
Ten day plaque assay results: (**A**) images of three replicates per strain (arrow: individual plaques formed by EQ40 were no longer distinguishable after fusion); (**B**) mean number of plaques (* *p* < 0.05; ** *p* < 0.01; average ± SEM: K1 228.5 ± 29.05; BGD18 (ToxoDB#1) 139.8 ± 8.6; EQ39 (ToxoDB#54) 105.5 ± 5.47; G13 93.6 ± 6.2).

The in vitro invasion kinetics analysis showed that of the lineage III strains, G13 exhibited the longest invasion time, as infected cells were observed after an incubation period of at least 3 h, whereas the other lineage III strains were able to invade cells within 1 h (Table 4). The lineage II archetype used as a control strain (BGD18) had an invasion time of approximately 1.5 h in these assays, but as the invasion time of G13 was at least 3 h, only the 1 h and 3 h time points are shown. Proliferation assays revealed that tachyzoites of the intermediately virulent strains EQ40 and K1, had a higher growth rate than the low virulence strain EQ39 (ToxoDB#54), while G13 displayed a fairly high mean number of divisions of within 48 h (Table 5). Concordantly, K1, EQ39 and BGD18 had significantly fewer tachyzoites per vacuole than EQ40 (*p* = 0.0282, *p* = 0.0086, and *p* = 0.0025, respectively; t-test) (Figure 3). G13 also contained fewer tachyzoites per vacuole than EQ40, albeit not significantly. The 10 day plaque assays showed that the plaque formed by EQ40 covers the largest area, the result of fusion of individual plaques over time (Figure 4A), and thus individual plaques could not be counted. A high variability in plaque formation was shown among the other examined isolates, as K1 formed the highest number when compared to EQ39 and G13 (*p* = 0.0019 and *p* = 0.0011, respectively, by t-test) (Figure 4B).

**Figure 5:**
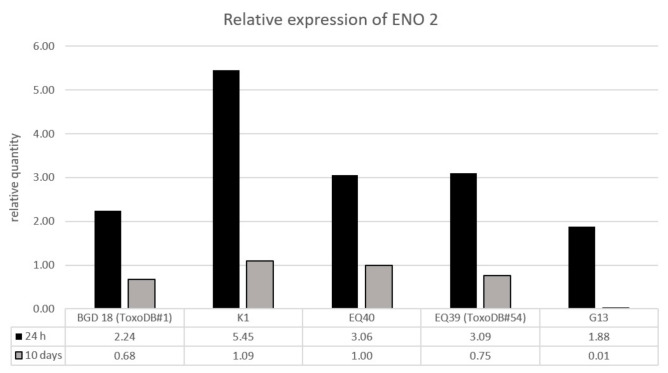
Relative quantification (ΔΔCt) of ENO2 after 24 h and 10 days of in vitro culture.

Relative quantification of the expression levels of ENO2 showed that after 24 h, the intermediately virulent isolate K1 expressed the highest level of the enzyme, the other intermediately virulent strain EQ40, and the low virulence strain EQ39 (ToxoDB#54) expressed similar levels, while G13 expressed the lowest level (Figure 5). The ENO2 expression level in BGD 18 (ToxoDB#1) was lower than in K1, EQ40, and EQ39. ENO2 expression after 10 days was generally lower in all tested isolates than at the 24 h time point, however the levels were higher in the two intermediately virulent strains, K1 and EQ40, than in the low virulence ones, EQ39 (ToxoDB#54) and G13. In the latter, ENO2 expression was barely detectable. ENO2 expression in strain BGD18 (ToxoDB#1) was lower than in the intermediately virulent strains and in EQ39 (ToxoDB#54) (Figure 5).

## 4. Discussion

This is the first report of MnPCR-RFLP genotypes using conventional markers altSAG2, BTUB, GRA6, C22-8, C29-2, L358, PK1, CS3, and Apico of all four lineage III isolates from Serbia, while EQ39 (ToxoDB#54), EQ40, and G13 were previously genotyped by microsatellite (MS) markers [19]. A search of ToxoDB revealed that genotype #54 was previously isolated from two sea otters and one white-tailed deer in North America (www.toxodb.org/toxo/app). Based on MS marker analysis, the genotype of strain EQ40 has been previously isolated from a goat in Gabon (TgA105011) [33], a pig in Portugal (TgPiPr14) [34], and a sheep from France (TgA32122) [35]. Interestingly, genotypes ToxoDB#54 and EQ40 are distributed over two continents each, Europe and North America and Europe and Africa, respectively, while the distribution of G13 and K1, which we were unable to genotype match to other ToxoDB isolates or in the MS database, is at present limited to Serbia. In fact, the genotype of strain K1 was likely also detected in a red fox [21], suggesting that it may circulate sylvatically and domestically, while G13 is the sole representative of that genotype based on current knowledge. In vivo virulence estimation demonstrated that the Gabon isolate TgA105011 did not induce mortality of infected mice, while infections with both EQ40 and TgPiPr14 resulted in mortality within 15 and 9–12 days, respectively, indicating virulence. There are no reports on mouse mortality induced by the French isolate, which originated from a sheep from the Pyrenees region on the Spanish border, geographically closest to Portugal. Infections of laboratory mice with TgA105011, EQ40, and TgPiPr14, conducted according to the same experimental protocol (making the data comparable), showed a dramatic discrepancy between high mortality elicited by the two European isolates and none by the African. Since all four isolates were genotyped by MS, one explanation may be that these are not actually identical but merely similar genotypes, as there may be additional polymorphisms at loci not analyzed by canonical MS markers, a possibility already proposed to explain the differences in virulence of two other strains of the same genotype analyzed by Hamilton et al. [36]. While the former is possible, it is more probable that these are indeed identical genotypes, but that the differences in virulence are attributable to different allele combinations of particular genetic virulence markers, as shown to be the case for a number of strains of different degree of virulence but with identical MnPCR-RFLP genotypes [37]. In fact, Shwab et al., 2016 [37] have demonstrated that select combinations of ROP5 and ROP18 alleles can be highly predictive of virulence. The combination ROP5^3^/ROP18^3^, which occurs quite frequently and is present in all lineage III isolates examined here, appears to have the least predictive virulence phenotype, as supported by the cumulative mortalities shown in Table 2. Thus, insight into the MnPCR-RFLP genotypes and the ROP5/ROP18 allele combinations of the African (TgA32122) and Portuguese (TgPiPr14) strains, which have not been published, could possibly provide an explanation for the discrepant mortality results. The results obtained here, however, do not preclude a genetic foundation for virulence but rather suggest that a greater number of known and potential virulence marker alleles needs to be examined. In contrast to EQ40, the results of the in vivo virulence of EQ39 (ToxoDB#54) and allele analysis of ROP5 and ROP18 were in concordance with previously published findings, which have determined this to be a genotype of low virulence [24,37]. As we were unable to match the MnPCR-RFLP genotypes and/or the MS genotypes of G13 and K1 with any known isolates, this represents the first report on their virulence in vivo as well as virulence marker allele analysis.

The virulence estimated in vivo generally agrees with the results of the in vitro assays for all examined isolates. Both intermediately virulent genotypes exhibit rapid cell invasion and enhanced intracellular multiplication and induce greater host cell death within 10 days as compared to the isolates of low virulence. Vacuole loading was significantly different between EQ40 and the other strains after 48 h in culture (Figure 3), however the plaque assay results were the most striking (Figure 4A). The plaque size (in case of EQ40) and number (in case of K1) correlate well with the high in vivo mortality rates up to day 15 post infection. As plaque area measurement for EQ40 could not be performed, an attempt was made to include the strain in the statistical calculations by quantifying the plaques after a 5 day incubation period (data not shown); however, the number of plaques formed by the low virulence strains G13 and EQ39 (ToxoDB#54) in that time interval was too low to allow for statistical comparison. Nonetheless, the number of plaques formed after 10 days by the intermediately virulent strain K1 was significantly higher than that formed by EQ39 (ToxoDB#54) and G13 (*p* = 0.019 and *p* = 0.011, respectively) (Figure 4B). The slow invasion exhibited by strain G13 (Table 4) may point to a defect in the invasion process itself, which could explain the low virulence despite a fairly high proliferation rate. In fact, G13 consistently infected visibly fewer cells per well, resulting in fewer vacuoles and higher SEM, than all the other strains, even when increasing the MOI, which does seem to support this explanation. A more extensive analysis of the invasion process of this strain is warranted. While proliferation rate is a plausible virulence mechanism, the rate clearly needs to be sustained over time and supported by high rates of re-invasion of newly egressed tachyzoites to ultimately produce the plaque assay results exhibited by EQ40 and K1. Proliferation rate, invasion, and host cell lysis have been previously discussed as likely mechanisms of virulence of archetype I [38]. As the invasion kinetics assays have shown that all strains but G13 invade cells within 1 h, it stands to reason that the re-invasion rate for all strains is similar, suggesting that a sustained high proliferation rate over time is crucial for virulence—case in point seems to be archetype I, which is dose-independently lethal to mice after in vivo infection. In addition, Saeij et al. [38] and others have suggested that in vitro proliferation rates of certain strains may decrease over time, which certainly seems likely, due to the limitations inherent to in vitro culture itself. However, it is interesting to note that these changes have been observed with genotypes of low virulence and not archetype I [38].

Our data indicate that the expression level of the tachyzoite specific enolase ENO2 is higher in intracellular tachyzoites of the intermediately virulent strains K1 and EQ40, than in the avirulent strains, EQ39, G13, and BGD18 after 10 days of in vitro culture (Figure 5). As ENO2 is more processive than the bradyzoite specific enolase ENO1, it has been speculated that ENO2 contributes to and/or facilitates the higher metabolic rate required by the tachyzoite life stage of the parasite during which it rapidly proliferates [26,39]. Thus, it is possible that in 10 day cultures formation of a large plaque area by the intermediately virulent strain EQ40 and significantly more plaques by K1 in comparison to the low virulence strains EQ39 (ToxoDB#54) and G13, as well as BGD18, is a result of a higher level of ENO2 expression, which likely supports higher proliferation rates. The results indeed point to the conclusion that a sustained high proliferation rate supports virulence. Interestingly, the results also showed that intracellular tachyzoites of low virulence strains G13 and BGD18 express lower levels of ENO2 after 24 h than the intermediately virulent strains K1 and EQ40. EQ39 (ToxoDB#54) was an exception, as the levels of ENO2 were similar to EQ40 after 24 h, suggesting high metabolic activity and possibly a fairly high rate of proliferation of EQ39 (ToxoDB#54) at least at that early time point. As the levels of ENO2 were determined in intracellular tachyzoites, the exceptionally low level exhibited by G13 after 24 h may again point to a defect in cell invasion. The data furthermore demonstrate that the metabolic rate of all tested strains is indeed attenuated over time, as expected in vitro, evidenced by the downregulation of ENO2 at day 10.

## 5. Conclusions

This study showed that virulent lineage III strains are present and circulate in the human food web in Serbia. Presence of virulent type III strains has previously been reported in Portugal [34]. Moreover, one intermediately virulent strain described here, EQ40, is likely identical to the Portuguese TgPiPr14, suggesting this strain is endemic, but as this genotype has not yet been isolated from or detected in human materials in either country, its clinical impact remains unknown. 

The study has also highlighted the importance of examining the growth phenotype of a particular parasite strain, as proliferation and plaque formation in vitro correlate with in vivo virulence. The mechanisms of virulence appear to include a higher metabolic rate, facilitated by ENO2, which sustains higher proliferation rates over time. Further research of ENO2 expression as a potential determinant of *T. gondii* virulence is warranted. To the extent to which the results of in vitro assays may be extrapolated to in vivo infections, the delayed metabolic attenuation in more virulent strains demonstrated in vitro may suggest delayed stage conversion to bradyzoites in vivo.

## Figures and Tables

**Table 1:** MnPCR-RFLP genotypes of the lineage III isolates. EQ39 matches ToxoDB#54, while the other isolates could not be matched in ToxoDB.

Isolate ID	Alt. SAG2 (Chr VIII)	BTUB (Chr IX)	GRA6 (Chr X)	C22-8 (Chr Ia)	C29-2 (Chr III)	L358 (Chr V)	PK1 (Chr IV)	CS3 (Chr VIIa)	APICO (Plastid)
EQ40	II	I	III	II	III	III	II	III	III
K1	II	II	I	II	III	III	III	III	III
EQ39 (ToxoDB#54)	II	III	III	III	III	III	III	II	II
G13	III	III	III	II	III	III	II	III	III

**Table 2:** Cumulative mortality and virulence determination.

Isolate ID	Cumulative Mortality (%) at Day 42	Virulence Classification [32]
EQ40	69.4%	Intermediately virulent
K1	38.8%	Intermediately virulent
EQ39 (ToxoDB#54)	6.8%	Non-virulent
G13	10.7%	Non-virulent

**Table 3:** Virulence marker allele typing.

Isolate ID	ROP5	ROP16	ROP18	GRA15
EQ40	3	1	3	3
K1	3	1	3	3
EQ39 (ToxoDB#54)	3	1	3	3
G13	3	1	3	3

**Table 4:** In vitro invasion kinetics (+ denotes the presence of infected cells, − denotes absence).

Strain	Time Post Infection	
	1 h	3 h
RH (ToxoDB#10)	+	+
BGD18 * (ToxoDB#1)	−	+
EQ40	+	+
K1	+	+
EQ39 (ToxoDB#54)	+	+
G13	−	+

* Strain BGD18 is a lineage II archetype isolated in house from cord blood of a congenitally infected neonate.

**Table 5:** In vitro proliferation parameters.

Strain	Mean Growth Parameters 48 h Post Infection			
	No. of Parasites/Vacuole	SEM	No. of Divisions (log_2_ of Parasites/Vacuole)	SEM
RH (ToxoDB#10)	27	4.250	3.712	0.476
BGD18 (ToxoDB#1)	3.6	0.880	0.981	0.244
EQ40	7.7	0.610	2.008	0.191
K1	5.4	0.436	2.025	0.127
EQ39 (ToxoDB#54)	4.9	0.611	1.625	0.157
G13	5.0	1.550	2.033	0.515
